# Influence of High-Frequency Ultrasonic Vibration Load on Pore-Fracture Structure in Hard Rock: A Study Based on 3D Reconstruction Technology

**DOI:** 10.3390/ma17051127

**Published:** 2024-02-29

**Authors:** Jianguo Zhang, Lei Zhang, Xufeng Wang, Zhijun Niu, Zhanbiao Yang

**Affiliations:** 1 State Key Laboratory of Coking Coal Resources Green Exploitation, China Pingmei Shenma Group, Pingdingshan 467000, China; 05181552@cumt.edu.cn (J.Z.); 01180281@cumt.edu.cn (Z.Y.); 2 Jiangsu Engineering Laboratory of Mine Earthquake Monitoring and Prevention, School of Mines, China University of Mining and Technology, Xuzhou 221116, China; wangxufeng@cumt.edu.cn (X.W.); niuzhijun@cumt.edu.cn (Z.N.)

**Keywords:** high-frequency vibration, nuclear magnetic resonance, CT scanning, three-dimensional reconstruction

## Abstract

Rock fracture is a macroscopic fracturing process resulting from the initiation and propagation of microscopic cracks. Therefore, it is crucial to comprehend the damage and fracture mechanism of rock under ultrasonic vibration by investigating the evolutionary pattern of the meso-pore fracture structure in response to high-frequency vibrational loads, as explored in this study. Standard red sandstone samples with a diameter of 50 mm and height of 100 mm were subjected to ultrasonic high-frequency vibration tests. NMR and CT scans were conducted on the rock samples at different stages of ultrasonic vibration excitation to obtain the corresponding transverse relaxation time (T_2_) spectra and CT scan images for each layer. The NMR test results revealed that smaller pores formed within the rock under high-frequency vibration loads, with a noticeable expansion observed in micropores. Three-dimensional reconstruction analysis based on two-dimensional CT images demonstrated an increase in pore count by 145.56%, 122.67%, and 98.87%, respectively, for the upper, middle, and lower parts of the rock after 120 s of ultrasonic vibration excitation; furthermore, the maximum pore volume increased by 239.42%, 109.16%, and 18.99%, respectively, for these regions during this period as well. These findings contribute towards a deeper understanding regarding the mechanisms underlying rock fragmentation when exposed to high-frequency vibrational loads.

## 1. Introduction

Hard rock excavation engineering is the pivotal link in the process of underground resource exploitation, and the speed of rock breaking directly impacts both the efficiency and cost of resource extraction, as well as the economic benefits of a project. Particularly in complex stress environments at great depths, rocks are often hard to break using traditional mechanical methods, leading to difficulties in improving excavation efficiency. While drilling and blasting techniques for breaking hard rock formations are relatively mature, they still suffer from low energy utilization rates, limited mechanization potential, and high risk [1,2,3,4], making it challenging to meet safety requirements while achieving efficient underground rock engineering. Therefore, there is an urgent need for new technologies that can efficiently break through these challenges.

The high-frequency vibration-assisted drilling tool rock breaking method, characterized by its advantages of superior precision, efficiency, and energy conservation [5], has found applications in the processing of brittle materials, as well as space drilling [6,7,8], thereby presenting a novel approach for the efficient excavation of hard rocks underground. Notably, some researchers have introduced this method into the domain of oil drilling by integrating it with traditional rotary drilling techniques and establishing a high-frequency rotary impact test platform [9]. Through comprehensive testing and theoretical analysis, it was confirmed that the bit’s drilling efficiency under high-frequency vibration surpassed that of conventional methods by tenfold. Consequently, investigating the mechanism behind rock fragmentation under high-frequency vibration loads can offer valuable theoretical insights for the practical implementation of this technology.

Currently, there is a scarcity of relevant studies on this technology. Li conducted numerical simulations to analyze the rock intrusion process during drilling under high-frequency vibration, investigating changes in the rock stress field and displacement field. The findings revealed that high-frequency vibration loads accelerate rock damage and reduce its maximum static load-bearing capacity [10]. Additionally, Li developed a mechanical model to examine the response of rocks to the impact of high-frequency vibrations, demonstrating that the rock response is directly proportional to force, vibration frequency, and rock mass [11]. Zhao employed electron microscope scanning to analyze the microfracture characteristics of granite under ultrasonic vibration excitation, revealing that microcracks primarily expand due to tensile failure influences [12]. Yin investigated the influence of static load on the mechanical properties of granite under ultrasonic high-frequency vibration through uniaxial compressive tests, discovering an optimal static load causing a significant reduction in rock mechanical strength [13]. Zhou monitored axial and radial strain on granite samples’ surfaces during ultrasonic vibrations and categorized the rock failure process into three stages: elastic deformation, plastic deformation, and damage [14]. Using an infrared thermal imager, Zhao studied the thermal damage effect of granite under ultrasonic vibration, divided the rock region into an elastic zone, a plastic zone, and a failure zone according to the surface temperature of the rock sample, and proposed that the non-uniform expansion of mineral particles inside the rock and fatigue damage are the main causes of granite fracture [15]. Zhang analyzed the influence mechanism of static load on rock failure under ultrasonic vibration from the perspectives of displacement field, stress field, fracture field, and energy in combination with tests and numerical simulation [16]. Yang used the penetration depth of the actuator, the degree of fragmentation, and the number of cracks as three indexes to evaluate the rock-breaking effect of ultrasonic vibration [17]. The sensitivity of vibration parameters to the rock-breaking effect of vibration load was analyzed, revealing that the amplitude, frequency, and load surface size are the main factors affecting the three indexes, respectively. Combining the strain test results, Zhou proposed a rock fatigue damage model under ultrasonic vibration and programmed a particle flow program to truly simulate the cracking failure process of granite samples under high-frequency vibration [18].

In most of the above studies, the influence of a high-frequency vibration load on rock failure is analyzed from a macroscopic perspective, focusing on rock mechanical strength, surface temperature, strain and fragmentation degree, etc. However, the damage and fracture of rock is a process that develops from the initiation, expansion, and penetration of microscopic cracks to macroscopic cracks, so it is necessary to explain the rock failure mechanism from a microscopic perspective. In addition, most of the above studies take granite as the research object, and the lithology is single. In this pap brittle fine-grained red sandstone is taken as the test object, and the evolution law of rock microporous fracture structure under the excitation of ultrasonic high-frequency vibration is analyzed by the comprehensive use of CT scanning technology, nuclear magnetic resonance technology, and three-dimensional reconstruction technology.

## 2. Materials and Methods

### 2.1. Experimental Equipment

#### 2.1.1. Sample Preparation

**Figure 1:**
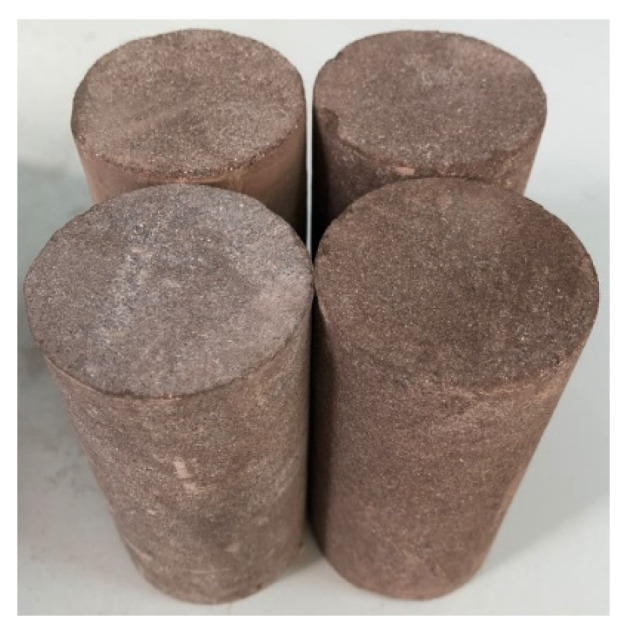
Rock samples.

The experimental subject of this study is brittle fine-grained red sandstone, sourced from Sichuan Province, China. The rock samples were prepared to meet the ISRM standard (International Society of Rock Mechanics) [19], with a standardized size of 50 mm in diameter and 100 mm in height, as depicted in Figure 1. Mechanical testing revealed an average compressive strength of the rock sample at 92.8 MPa and an elastic modulus of 5.2 GPa.

#### 2.1.2. Ultrasonic High-Frequency Vibration Test System

**Figure 2:**
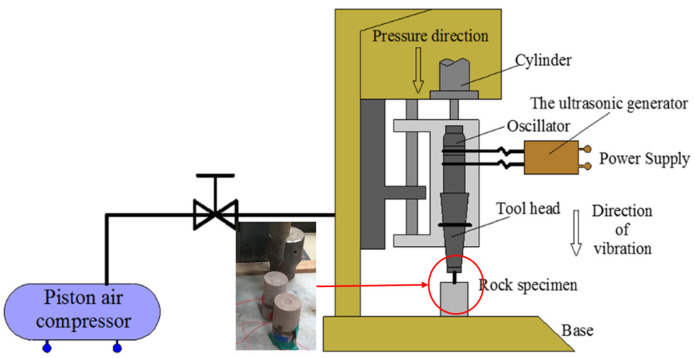
Ultrasonic high-frequency vibration excitation testing system.

The ultrasonic high-frequency vibration system is utilized for the application of vibrational loads onto the red sandstone sample. The development of this system was undertaken by Professor Wang’s research team at China University of Mining and Technology. Figure 2 illustrates the schematic diagram of the device employed in this study. The device utilizes an ultrasonic transducer to convert high-frequency alternating electrical signals generated by the ultrasonic generator into mechanical vibrations, which are then amplified through an amplitude transformer to achieve a magnitude capable of breaking the rock. A circular area with a central radius of 13 mm on the end face of the rock serves as the load surface, and static load is applied to ensure close contact between the actuator and the rock. In this research, a static load of 0.25 MPa was applied to the rock, while maintaining a fixed vibration frequency of 20 kHz and an amplitude set at 70 μm.

#### 2.1.3. Nuclear Magnetic Resonance System

The principle of utilizing nuclear magnetic resonance (NMR) technology for rock pore structure analysis is as follows: by subjecting the rock sample to a specific intensity magnetic field, the ^1^H atomic signal emitted by fluid within microscopic pores can be detected. Parameters such as the transverse relaxation time (T_2_) of the fluid provide insights into the rock’s pore structure. A higher *T*_2_ spectrum amplitude indicates a greater presence of hydrogen atoms in the fluid, allowing the estimation of pore volume based on the hydrogen atom count. Additionally, the transverse relaxation time reflects the size of rock pore diameter, and their relationship can be expressed as follows [20]:

(1) $$T_{2}=\frac{V}{\rho s}=\frac{r}{\rho c}$$

where the transverse relaxation time is represented by *T*_2_, the pore volume is denoted as *V*, the pore surface area is indicated by *s*, the relaxation strength is symbolized by *ρ*, the pore radius is represented by *r*, and the pore shape factor is denoted as *c*.

**Figure 3:**
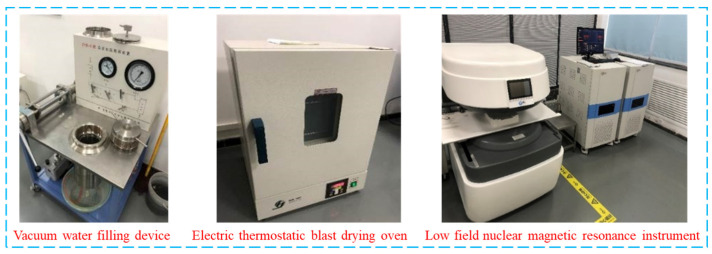
Nuclear magnetic resonance system.

In this study, the MiniMR12-15H-I nuclear magnetic resonance system was utilized to perform NMR tests on rock samples pre and post ultrasonic vibration excitation. The system boasts a power output of 2.5 kW, with a working voltage range of 220–380 V, and utilizes permanent magnet technology. Additionally, the main frequency reaches an impressive 12.8 MHz. The experimental setup comprised an electric thermostatic blast drying oven, a rock vacuum water filling device, and a low field nuclear magnetic resonance instrument (Figure 3). Specifically, the constant temperature blast drying oven had a power rating of 950 W and offered a temperature setting range of 10–300 °C. The maximum core size of the vacuum water filling device was Φ120 × 400 (mm), with a water filling pressure range spanning from 0 to 60 MPa; moreover, it could achieve a vacuum pressure as low as 0.1 MPa.

#### 2.1.4. Computed Tomography (CT) Scanning System

**Figure 4:**
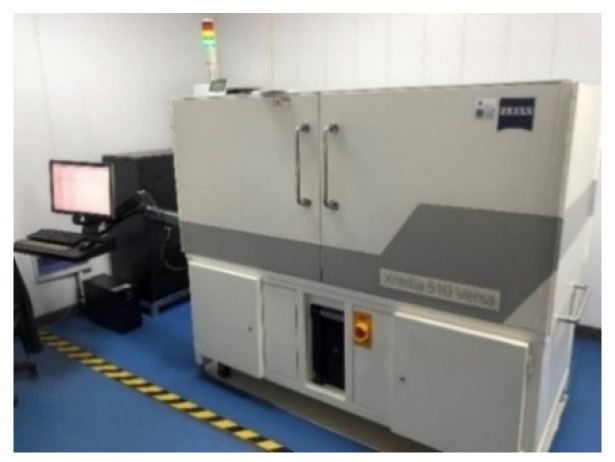
High resolution 3D X-ray microscopy imaging system.

CT scanning technology, as a non-destructive technique for obtaining the morphological characteristics of the meso-structure within rocks, enables the acquisition of structural features from each section. In this study, a high-resolution three-dimensional X-ray microscopic imaging system (3D-XRM) was employed to perform CT scanning, yielding tomography images with an impressive resolution of 1003 × 1024 pixels, as depicted in Figure 4. The system is capable of testing samples with a maximum diameter of 75 mm and a maximum height of 70 mm, utilizing X-ray sources ranging from 20 to 100 kV.

**Figure 5:**
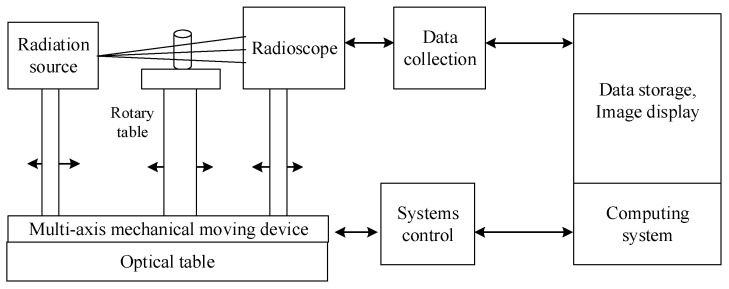
CT scanning principle.

The principle of rock CT imaging is illustrated in Figure 5. An X-ray beam is employed to perform a scan on the rock layer with a specific thickness. Subsequently, the received X-ray signal passes through the detector and undergoes digital–analog conversion, followed by computer processing to reconstruct a sectional image of the rock.

When X-ray is employed for rock scanning, different materials within the rock exhibit varying absorption capacities towards X-rays, resulting in distinct degrees of attenuation. The extent of attenuation can be represented as follows [21]:

(2) $$I=I_{0}e^{-\mu_{0}x}$$

where *I*_0_ and *I* represent the respective intensities of X-rays emitted by the source and received by the detector, while *x* denotes the thickness of the X-ray passing through the material, and Q signifies the attenuation coefficient of said material.

### 2.2. Experimental Procedures

#### 2.2.1. Nuclear Magnetic Resonance Test

**Figure 6:**
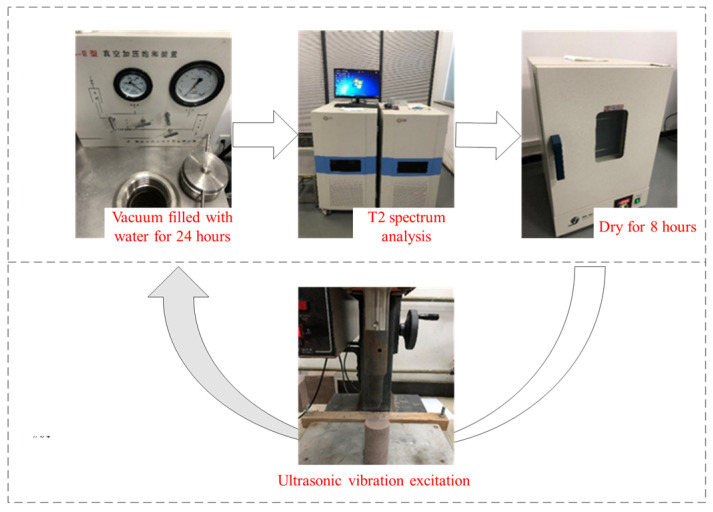
Procedure of NMR experiment.

Three standard cylindrical red sandstone samples (S1, S2, and S3) were selected for this study. The NMR experiment process for monitoring pore changes in a single rock sample is illustrated in Figure 6. Initially, the unstimulated rock sample was saturated with water, followed by NMR T_2_ spectral analysis. Each excitation time lasted for 40 s, and these steps were repeated until the rock sample fractured, or the test was terminated.

#### 2.2.2. CT Scanning Test

**Figure 7:**
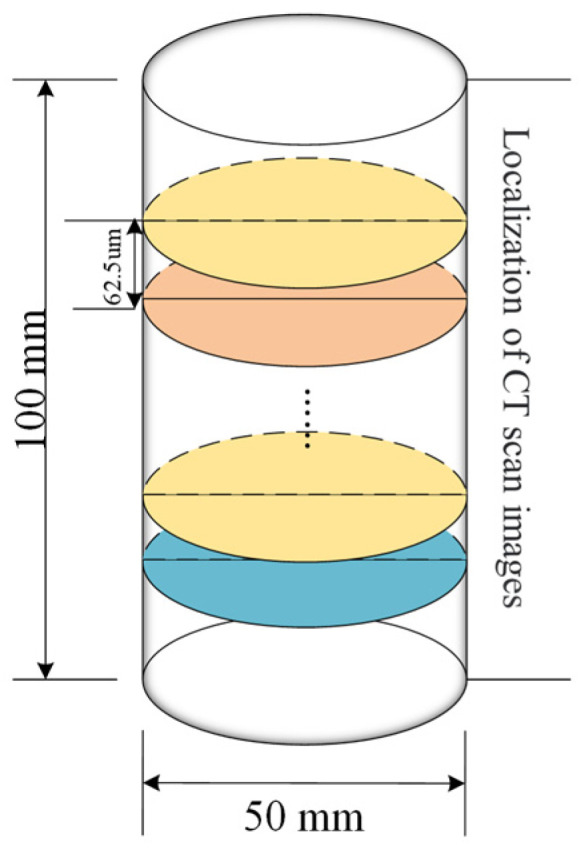
Scheme of CT scanning.

A cylindrical red sandstone sample was selected for CT scanning every 40 s. The scanning scheme is shown in Figure 7. The top end of the rock sample (excitation end) is scanned every 62.5 μm, and the rock sample yields about 1600 sectional images each time, which are used as the basis for subsequent processing and analysis.

## 3. Results

### 3.1. Evolution of NMR T_2_ Spectra of Rock Samples under Ultrasonic Vibration

**Figure 8:**
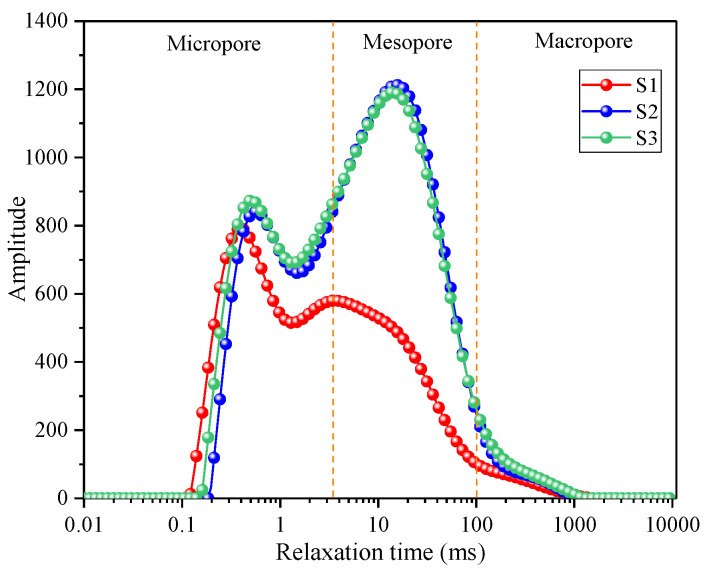
T_2_ spectrum of rock samples in their natural state.

Through the nuclear magnetic resonance experiment, the T_2_ distribution curves of the three samples in their initial states were obtained, as illustrated in Figure 8. The transverse relaxation time serves as an indicator of the pore diameter within the rock, which can be further classified into micro pores, medium pores, and large pores, corresponding to distinct regions on the T_2_ curve. The T_2_ distribution curves of the three rock samples exhibit similarities, displaying bimodal peaks that are situated in the micropore and mesopore regions, respectively. The order of peak designation from left to right is as follows: first peak and second peak. Sample S1 exhibits a slightly distinct pore structure compared to S2 and S3. Peak 1 of sample S1 surpasses peak 2, indicating a greater abundance of micropores relative to mesopores, suggesting a relatively dense rock matrix. The curves for S1 and S2 largely coincide, signifying highly similar pore structures, with a predominant contribution from mesopores. The initial porosity measured by NMR for the three samples was determined as 3.98%, 7.31%, and 7.82%, respectively, yielding an average value of 6.37%.

**Figure 9:**
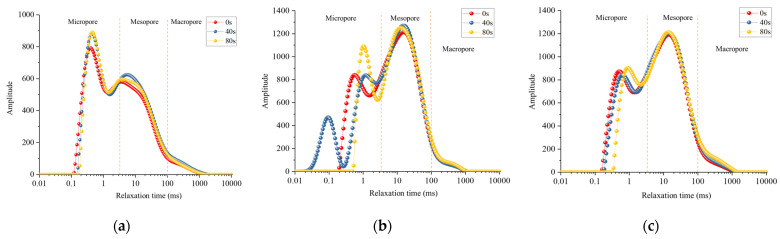
Evolution of T_2_ spectrum curves of three rock samples: (**a**) S1; (**b**) S2; and (**c**) S3.

The T_2_ distribution curves of samples S1, S2, and S3 under ultrasonic vibration stimulation are presented in Figure 9. Pore structure alterations primarily occur within the micropore region. After 40 s of ultrasonic vibration stimulation, peaks 1 and 2 of sample S1 shifted to the right while increasing in magnitude, indicating an expansion of micropores within the rock. A new peak emerged on the left side of the T_2_ curve for rock sample S2, suggesting the presence of smaller micropores. Simultaneously, peak 1 shifted to the right, signifying that the original micropores expanded, and some even extended into mesopores, slightly augmenting peak 2. Similarly, the internal micropores expanded in sample S3, while the mesopores remained mostly unchanged. Following cumulative vibration excitation for 80 s, minimal pore changes were observed in sample S1, with only a slight decrease observed in peak 2 due to the continuous expansion of certain mesopores. Rock porosity variations were evident in sample S2, as the newly generated peak vanished, along with an increase and displacement observed in peak 1—indicating expansion occurred within all newly formed rock micropores. Micropore expansion persisted within sample S3 during different stages of ultrasonic vibration excitation; however, there were relatively minor changes observed in peaks 2 among all three rocks. Particularly noticeable is the overlapping behavior among larger pores towards the latter part of each curve.

**Figure 10:**
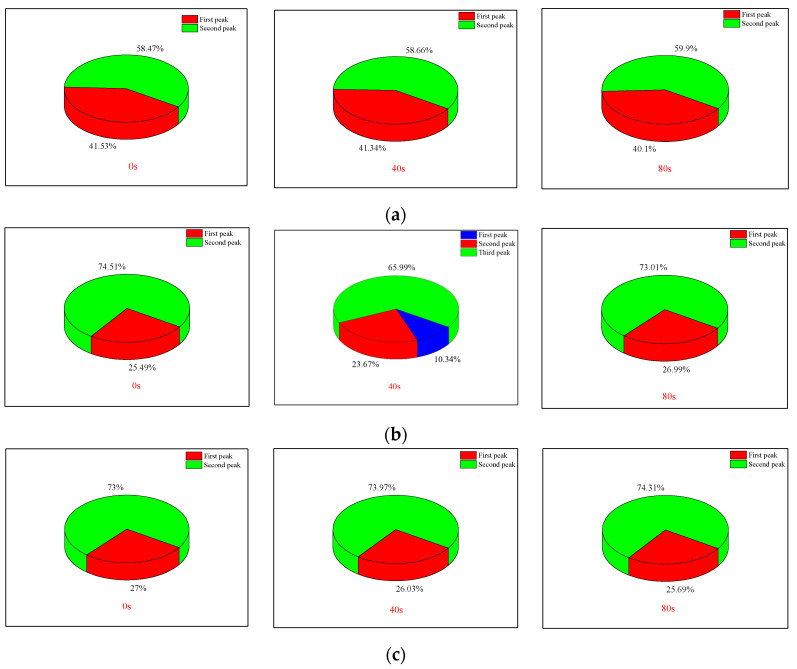
Proportion of each peak area of rock samples under different excitation times: (**a**) S1; (**b**) S2; (**c**) S3.

The changes in the proportion of each peak are illustrated in Figure 10. Following ultrasonic cumulative vibration excitation at 80 s, the area proportion of peak 2 in the T2 distribution curve increased by 1.5% and 1.31% for rock samples S1 and S3, respectively. Conversely, sample S2 exhibited a decrease of 1.5%, which can be attributed to the introduction of numerous micropores into the S2 rock sample. Based on this analysis, it is evident that ultrasonic vibration excitation effectively promotes micro-pore formation and expansion within rocks, while exerting minimal influence on large and medium-sized pores.

### 3.2. Three-Dimensional Pore Fracture Propagation Characteristics of Rock Based on 3D Reconstruction Technology

**Figure 11:**
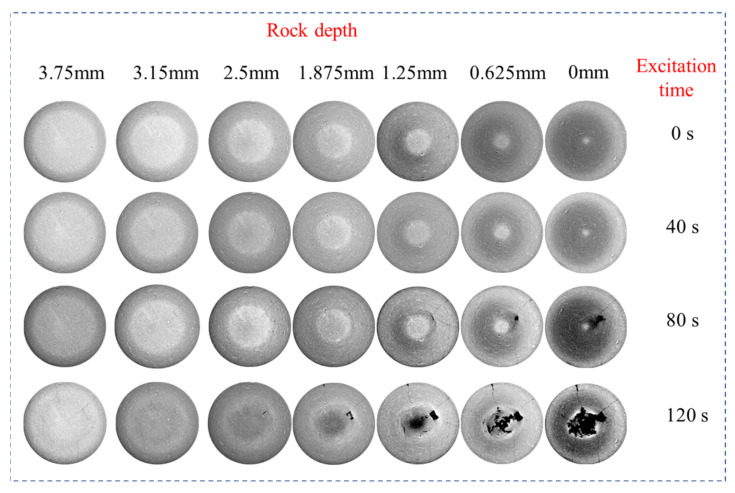
Microscopic crack distribution in rock.

The rock samples subjected to ultrasonic vibration loads did not exhibit fractures throughout the entire body, with failure only concentrated in the localized section surrounding the top load surface. Figure 11 illustrates the development of fractures within each layer in the typical failure range of rocks under different excitation stages. Initially, a fracture zone formed at the contact surface between the exciter and the rock, which then expanded continuously as microscopic cracks initiated and extended towards both lower regions and outer free planes. After 40 s of vibration stimulation, the maximum crack propagation depth reached 24.16 mm, increasing to 32.15 mm after 120 s of stimulation, representing a growth rate of 33%.

**Figure 12:**
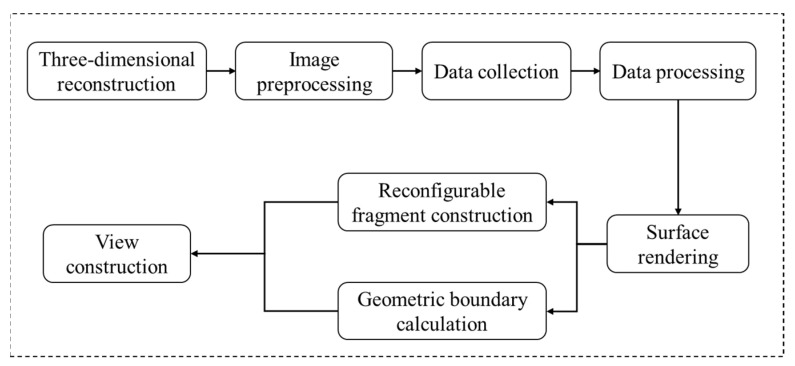
3D reconstruction procedure.

To further investigate the spatial expansion characteristics of internal pores and fractures under ultrasonic vibration loads, a three-dimensional data model of red sandstone was reconstructed, based on two-dimensional CT scan images. The three-dimensional reconstruction of CT scan images involves converting two-dimensional CT images into visually intuitive three-dimensional models, enabling an accurate representation of the geometric position and interrelationships among different parts of the model. This facilitates subsequent three-dimensional analysis and processing. The main steps involved in 3D reconstruction are illustrated in Figure 12.

**Figure 13:**
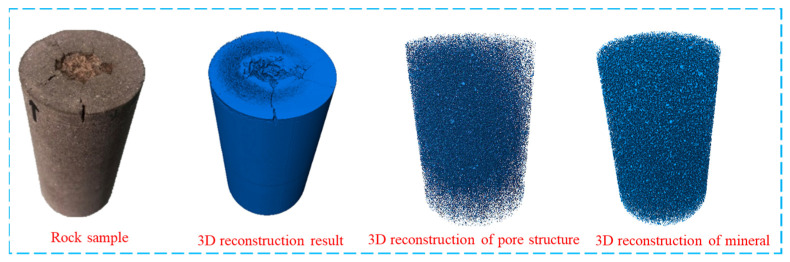
A 3D reconstruction of red sandstone.

The 3D reconstruction results are presented in Figure 13, showcasing a high level of consistency between the reconstructed rock and the actual samples in terms of overall morphology. Moreover, the visual representation exhibits exceptional quality, effectively illustrating the spatial distribution of macroscopic cracks on the rock surface.

**Figure 14:**
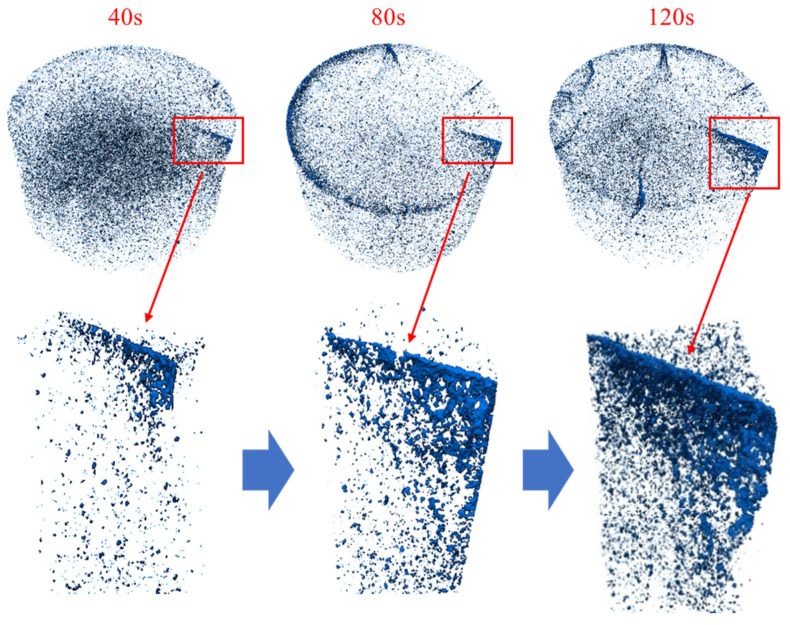
Three-dimensional fracture evolution process.

The cracks in the rock are predominantly concentrated in the upper region, as depicted in Figure 14, which showcases the three-dimensional reconstruction results of these cracks during different excitation stages. In its natural state, numerous minuscule pore groups are dispersed within the rock. However, when these pore groups aggregate and interconnect to form a distinct sheet-like structure, it signifies the presence of macroscopic cracks within the rock. The figure clearly exhibits the fracture surface inside the rock. Following the initial ultrasonic vibration excitation, a primary fracture surface emerges parallel to the axial direction. Subsequent excitations lead to a noticeable expansion of the extent of this fracture surface.

The expansion of the fracture surface can be attributed to two primary factors: firstly, stress concentration occurs at the crack tip, leading to crack propagation in the direction of applied stress; secondly, during the process of crack propagation, both pre-existing and newly formed pores within the rock continuously coalesce. Under vibrational excitation, new fracture surfaces are consistently generated. Following a second round of vibration excitation, two additional three-dimensional cracks emerge, while a third loading introduces three more cracks. Notably, the crack surface predominantly expands along the direction of vibrational loading.

**Figure 15:**
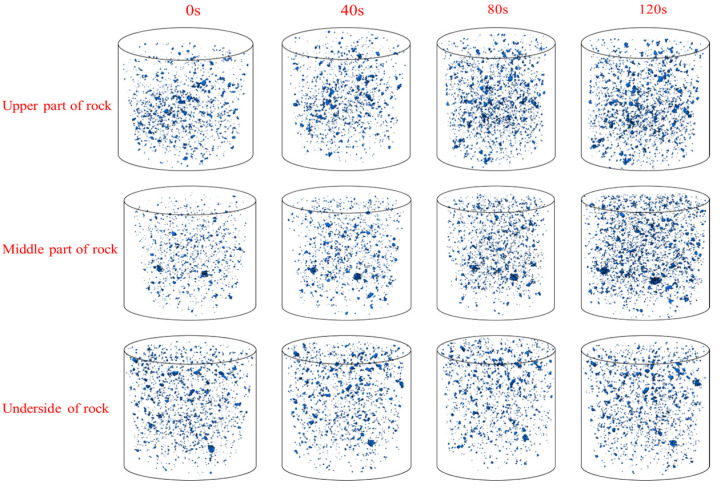
Three-dimensional pore evolution at different positions of rocks.

In order to visually observe the changes in three-dimensional rock pores, the rock sample was divided into three sections: upper (100–600 layers), middle (600–1100 layers), and lower parts (1100–1600 layers). A cylindrical region with a diameter and height of 160 pixels was extracted from the center of each section. The three-dimensional reconstruction of pores during different stages of vibration excitation is presented in Figure 15. Under ultrasonic vibration excitation, there is a significant increase in pore density within the rock, with a more pronounced increment observed closer to the upper part.

In the 3D reconstruction of pores, each pore is represented by a limited number of pixels, with larger pores containing more pixels. Consequently, the quantification of pores within the rock involves counting pixel combinations in the reconstruction, while the relative pore volume is depicted by the number of pixel points within each pore in the three-dimensional diagram. Pore sizes are categorized based on pixel count into ranges, such as 1–10, 11–20, 21–30, 31–40, 41–50, and above 50. The number of pores within different volume ranges was determined for an intercepted area of rock samples. Statistical results regarding pore counts in the upper, middle, and lower sections are presented in Table 1 (upper), Table 2 (middle), and Table 3 (lower). A comparison between pre-vibration excitation and post-ultrasonic vibration excitation, conducted thrice, revealed an increase in total pore numbers by approximately 145.56%, 76.89%, and 2.23%, respectively.

Based on the actual size conversion, the actual size corresponding to a single pixel is approximately 0.13852 μm^3^. The majority of pore volumes within the red sandstone are below 1.3852 μm^3^, with such pores accounting for an average proportion of 97.78% of all pores in their natural state within the rock sample. After being stimulated by ultrasonic vibration, there was a significant increase in the number of pores across all sizes inside the rock sample following three rounds of stimulation; specifically, there was a total increase in pore numbers at the upper part of the rock sample by 16,362 (a growth rate of 145.56%). Volume increases were observed for pores with sizes less than 1.3852 μm^3^ (133.29%), as well as for those ranging from 1.3852 to 2.7704 μm^3^ (554.6%), from 2.7704 to 4.1556 μm^3^ (501.67%), and from 4.1556 to 5.5408 μm^3^ (18 times). Pores sized between 5.5408 and 6.926 μm^3^ increased by 723.08%, while the number of pores with a volume greater than 6.926 μm^3^ increased by 754.17%. In comparison to these findings at upper levels, the middle and lower parts showed less pronounced increases after three rounds of loading: specifically, pore numbers increased by 122.67% and 98.87%, respectively.

The influence of ultrasonic vibration energy increases as one moves closer to the upper part of the rock, resulting in easier generation of new micropores within and a higher degree of fragmentation in this region. The propagation of vibration energy gradually attenuates downwards, with a minimal impact on the lower part of the rock and consequently negligible changes in pore number. Under ultrasonic vibration excitation, rock particles experience high-frequency tensile and compressive stress, while local tensile stress at grain boundaries within red sandstone primarily contributes to the formation of new pores. As vibration energy attenuates further away from the excitation position, rocks located at a distance undergo reduced stress levels, making it challenging for new pores to form, thus resulting in a limited number of newly formed pores.

**Figure 16:**
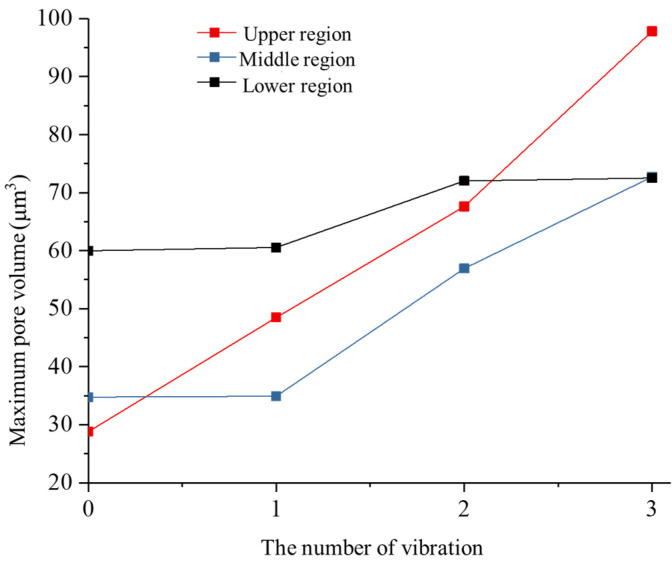
Evolution of maximum pore volumes at different rock locations.

According to the statistical analysis of the size of maximum pore mass within the rock sample, Figure 16 illustrates the change curve of maximum pore mass volume with vibration excitation time. In the absence of vibration excitation, the maximum pore volumes for the upper, middle, and lower samples are 28.81216 μm^3^, 34.76852 μm^3^, and 59.97916 μm^3^ respectively. Following the initial vibration excitation, only the upper part of the sample experienced an increase in maximum pore volume to 48.482 μm^3^, while no changes were observed in both middle and lower parts. After three rounds of vibration stimulation, all three parts exhibited increased pore volumes, with the most significant growth observed in the upper part. The maximum pore volume increased by 239.42% and 109.16% for the upper and middle parts, respectively; however, there was only a modest increase of 18.99% in the pore volume in the lower part compared to its initial value without vibrations applied. The change curve for maximum pore volume in lower part displayed a gentler slope, indicating less influence from ultrasonic vibration loading on pores within this region. Except for an increasing rate of growth observed specifically in the volumetric expansion of the upper part’s pores, the rest of the rock showed decreasing rates as time progressed.

## 4. Discussion and Conclusions

The relevant experimental studies primarily analyze the mechanical properties, macroscopic deformation principles, and failure mechanisms of rocks under ultrasonic vibration loads, from a macroscopic perspective. Macroscopic rock fractures are caused by the accumulation, development, and expansion of mesopore fissures within the rock. This paper investigates the impact of ultrasonic vibration loads on the meso-structure of rocks. Through various non-destructive detection techniques, the three-dimensional expansion characteristics of internal pores and cracks in rocks are qualitatively and quantitatively analyzed, thereby enhancing our current understanding of rock fragmentation through ultrasonic vibrations.

In this study, we conducted an ultrasonic high-frequency vibration excitation test on brittle fine-grained sandstone. Additionally, nuclear magnetic resonance (NMR) testing and CT scanning were performed on rocks at different excitation stages to investigate the impact of high-frequency vibration loads on the meso-pore fracture structure of hard rock. Based on these findings, three-dimensional reconstruction technology was employed to quantitatively analyze the expansion characteristics of three-dimensional pore fractures in rocks. The following conclusions were drawn:

(1) By comparing NMR T_2_ spectra of rocks at various vibration loading stages, it was observed that peaks in the micropore region shifted towards higher values, and new peaks appeared in some rock samples. This indicates that ultrasonic vibration loads can facilitate pore initiation and promote micropore expansion in rocks. Conversely, medium and large pores are less affected by vibration loads.

(2) CT scan images revealed that under ultrasonic high-frequency vibration excitation, a failure zone initially formed at the contact surface between the exciter and the rock. This region continued to expand, as microscopic cracks initiated downwards and around the rock. Compared to initial vibration excitation, after 120 s of stimulation, the depth of expansion reached 32.15 mm—a 33% increase.

(3) Three-dimensional reconstruction allowed us to obtain the crack morphology inside rocks during different excitation stages. It was found that crack surfaces predominantly expanded along the direction of vibrational loading. Comparing pre-vibration stimulation with post-120 s ultrasonic stimulation, there was a respective increase in total pore number for the upper (145.56%), middle (76.89%), and lower parts (2.23%) of the rock; the maximum pore volume also increased by 239.42%, 109.l6%, and 18.99%.

## Figures and Tables

**Table 1:** Pore distribution in upper section under different excitation times.

Time/s	Pore Size Distribution (Number of Pixels)						
	1–10	11–20	21–30	31–40	41–50	>50	Total Quantity
0 s	10,950	185	60	9	13	24	11,241
40 s	15,920	358	80	30	24	40	16,452
80 s	21,337	728	168	87	37	80	22,437
120 s	25,545	1211	361	174	107	205	27,603

**Table 2:** Pore distribution in central section under different excitation times.

Time/s	Pore Size Distribution (Number of Pixels)						
	1–10	11–20	21–30	31–40	41–50	>50	Total Quantity
0 s	10,690	142	50	15	9	15	10,921
40 s	11,778	162	55	21	9	16	12,041
80 s	14,621	299	74	38	18	37	15,087
120 s	18,262	680	189	81	43	63	19,318

**Table 3:** Pore distribution in lower section under different excitation times.

Time/s	Pore Size Distribution (Number of Pixels)						
	1–10	11–20	21–30	31–40	41–50	>50	Total Quantity
0 s	10,030	121	40	20	4	13	10,228
40 s	10,091	124	41	20	8	13	10,297
80 s	10,112	227	43	22	9	14	10,427
120 s	10,131	231	43	26	11	14	10,456

## Data Availability

Data are contained within the article.
